# How Do Quantitative Videofluoroscopy Measures Differ Between People With Amyotrophic Lateral Sclerosis and Age-Matched Healthy Adults?

**DOI:** 10.1044/2024_JSLHR-24-00106

**Published:** 2024-07-15

**Authors:** Pooja Gandhi, Ashley A. Waito, Melanie Peladeau-Pigeon, Emily K. Plowman, Catriona M. Steele

**Affiliations:** a Swallowing Rehabilitation Research Laboratory, KITE Research Institute–University Health Network, Toronto, Ontario, Canada; b Rehabilitation Sciences Institute, Temerty Faculty of Medicine, University of Toronto, Ontario, Canada; c Department of Speech-Language Pathology, Faculty of Health Sciences, McMaster University, Hamilton, Ontario, Canada; d Aerodigestive Research Core, University of Florida, Gainesville; e Department of Otolaryngology–Head and Neck Surgery, Division of Laryngology, The Ohio State University, Columbus; f Canada Research Chair in Swallowing and Food Oral Processing, Canada Research Chairs Secretariat, Ottawa, Ontario, Canada

## Abstract

**Purpose:**

Dysphagia is a leading cause of morbidity in people with amyotrophic lateral sclerosis (PwALS). Previous videofluoroscopic swallowing studies (VFSS) in PwALS do not account for the influence of senescence. We aimed to compare swallowing in PwALS and an age- and sex-matched control group using healthy reference data to define typical and atypical values.

**Method:**

We conducted retrospective analysis of VFSS data from 19 PwALS (10 male, *M*_age_ = 63 years, range: 47–82) compared to control data from a cohort of healthy adults. Participants swallowed 20% w/v liquid barium from thin to extremely thick consistency. Blinded duplicate VFSS analysis using the ASPEKT (Analysis of Swallowing Physiology: Events, Kinematics and Timing) method yielded descriptive statistics for 16 quantitative VFSS parameters by consistency. Mann–Whitney *U* tests were used to identify significant cohort differences. Additionally, the frequencies of atypical values (in the 25% tails of the reference distribution) were tabulated by cohort and compared using odds ratios.

**Results:**

PwALS showed increased frequencies of multiple swallows per bolus, incomplete laryngeal vestibule closure, and reduced hyoid speed across consistencies. By contrast, similar frequencies of atypical values for pharyngeal constriction and residue in both cohorts suggest that age-related changes may contribute to the presence of these features in PwALS.

**Conclusions:**

This analysis builds on previous descriptions of swallowing pathophysiology in amyotrophic lateral sclerosis (ALS) by clarifying the extent to which aging may account for some of the atypical findings seen in this patient population. Longitudinal studies are recommended to further differentiate the effects of ALS from age-related changes in swallowing over the course of disease progression.

Dysphagia occurs commonly in people with amyotrophic lateral sclerosis (PwALS) and involves changes across the oral, pharyngeal, and esophageal stages of swallowing, which contribute to impairments of swallowing safety (i.e., airway protection) and efficiency (i.e., bolus clearance; Brandão et al., 2018, 2019; Easterling et al., 2013; Garand, Bhutada, et al., 2022; Higo et al., 2004; B. H. Lee et al., 2019; J. Lee et al., 2021; Park et al., 2022; Robison et al., 2022; Ruoppolo et al., 2013; Waito et al., 2020). The most detailed descriptions of oropharyngeal swallowing in PwALS come from a series of papers arising from a large natural history study, which included data collection using a standard videofluoroscopy protocol at the Aerodigestive Research Core at the University of Florida (PI: Plowman; Donohue et al., 2023; Robison et al., 2022, 2023). Collectively, these papers profile swallowing impairment that follows a rostrocaudal progression, in which impaired swallowing efficiency, characterized by a pattern of multiple swallows per bolus and pharyngeal residue, precedes the emergence of impaired airway protection. Reduced tongue strength and reduced speed and range of tongue motion have been shown to predict impairments both in swallowing efficiency and safety (Perry et al., 2021; Robison et al., 2022, 2023). However, in order to identify and manage amyotrophic lateral sclerosis (ALS)–related dysphagia appropriately, it is important for clinicians to be able to differentiate changes in swallowing in ALS from those seen in healthy aging (Garand, Bhutada, et al., 2022, 2023; Kendall & Leonard, 2001a, 2001b, 2001c; Mancopes et al., 2021). This is important across the full range of bolus consistencies, given that bolus consistency is known to influence swallowing safety, efficiency, timing, and kinematics (e.g., Barbon et al., 2022; Barbon & Steele, 2015; Bolivar-Prados et al., 2023; Garand et al., 2024; Ito et al., 2020; Lenell et al., 2020; Leonard et al., 2014; Nagy et al., 2015; Newman et al., 2016; Park et al., 2022; Peña-Chávez et al., 2023; Sia et al., 2018; Smaoui et al., 2021; Steele et al., 2015, 2019; Wallace et al., 2023).

Our laboratory is currently engaged in a program of research, which aims to profile swallowing in adults without dysphagia (henceforth, “healthy adults”) and in different clinical cohorts, based on quantitative measures from videofluoroscopic swallowing studies (VFSS). We have developed both a standard data collection protocol and a standard operating procedure for VFSS rating known as the ASPEKT (Analysis of Swallowing Physiology: Events, Kinematics and Timing) method (Steele et al., 2019, 2023). A first aim of this research program has been to establish reference values for healthy swallowing that can be used to guide the clinical identification of parameter values that fall outside the range expected in healthy swallowing. In 2019, we published initial reference values for a sample of healthy adults aged under 60 years (under-60; Steele et al., 2019). Recently, we have published updated reference values for an expanded sample of healthy adults aged 21–82 years (Steele et al., 2023). A second aim is to describe swallowing in groups of patients with conditions in which dysphagia is common, including PwALS. In a previous study, we reported preliminary results for a sample of 19 adults with ALS (Waito et al., 2020), of whom 14 (74%) had unsafe swallowing on at least one bolus (Penetration–Aspiration Scale [PAS] score ≥ 3; Rosenbek et al., 1996) and 10 (53%) had inefficient swallowing on at least one bolus in the form of residue or multiple swallows. That analysis included a comparison of quantitative measures of thin liquid swallowing to values from the under-60 healthy reference sample (Steele et al., 2019). Findings in the direction of clinical concern in PwALS on thin liquids included significantly increased frequencies of partial or incomplete laryngeal vestibule closure (LVC), prolonged time-to-LVC, narrow upper esophageal sphincter (UES) distension, and poor pharyngeal constriction. Additionally, PwALS showed significantly longer durations of LVC and UES opening; these findings are in the opposite direction to findings of potential clinical concern and suggest possible compensations.

In the time that has elapsed since the Waito et al. (2020) publication, we have completed rating of the full data set from PwALS across multiple liquid consistencies (IDDSI Level 0 thin [TN0], Level 1 slightly thick [ST1], Level 2 mildly thick [MT2], Level 3 moderately thick [MO3], and Level 4 extremely thick [EX4]; Cichero et al., 2017), and we have acquired additional reference data, expanding the healthy reference cohort to a group of 78 adults aged 18–82 years (Steele et al., 2023). In 2022, we published an analysis of swallow timing, comparing latencies from hyoid burst onset (HYB) to other key events in the swallowing sequence in PwALS, together with data from a cohort of participants with Parkinson's disease, and the expanded healthy reference sample (Gandhi et al., 2023). This analysis identified significantly prolonged latencies from HYB in PwALS across consistencies for all key events, that is, bolus passing ramus of mandible (BPM), LVC, UES opening, maximum UES distension, maximum pharyngeal constriction (MPC), UES closure, LVC offset, and swallow rest.

These prior analyses do not control for the fact that the participants in our PwALS cohort were older, on average (*M*_age_ = 62 years, range: 47–78), than both the original reference sample (*M*_age_ = 34 years, range: 21–58) and the expanded reference sample (*M*_age_ = 51 years, range: 21–82). Swallowing is known to change as a function of healthy aging (Garand, Beall, et al., 2022; Garand et al., 2023; Kendall & Leonard, 2001a, 2001b, 2001c; Mancopes et al., 2021). Analysis of the thin liquid data in the full Steele et al. (2023) reference data set showed significant correlations between advancing age and increases in swallow reaction time (i.e., the latency from HYB to BPM), UES opening duration, LVC duration, and pharyngeal area (both at rest and at maximum constriction) and UES distension (Mancopes et al., 2021). Therefore, in order to control for the possible contributions of older age to the findings in PwALS, this article presents an analysis of the data from PwALS compared to age- and sex-matched controls from the expanded healthy reference sample. Specifically, we aim to answer the following research questions:

1. Do VFSS parameter values in PwALS differ significantly from values in healthy age- and sex-matched controls for each liquid consistency tested?
2. Compared to age- and sex-matched controls, do PwALS show an increased frequency of VFSS values of potential clinical concern (henceforth, “atypical”) by consistency? Here, we define atypical values as values falling beyond clinical decision limits set either at the 75th percentile of the healthy reference distribution (for parameters where larger values indicate values of potential clinical concern) or at the 25th percentile (for parameters where smaller values are of potential clinical concern).

Based on these analyses, we aim to clarify the nature of consistency-specific differences in videofluoroscopy parameters that might be considered characteristic of ALS. Due to the exploratory and descriptive nature of this study, we did not have specific a priori hypotheses beyond the null hypothesis that group differences would not be seen between PwALS and age-matched controls.

## Method

The original study protocols received human subjects ethical approval from the local institutional review boards at the ALS data collection site in Florida (IRB201701608) and at the healthy control data collection and data analysis site in Toronto (CAPCR ID 15-9431) and were registered at ClinicalTrials.gov (NCT03192358 and NCT04114617, respectively). The data collection methods in PwALS and in the healthy controls have been previously described by Waito et al. (2020) and Steele et al. (2023), respectively. Briefly, for both samples, we collected and analyzed quantitative videofluoroscopy data for swallows of thin, slightly thick, mildly thick, moderately thick, and extremely thick barium. The videofluoroscopies were performed using pulsed fluoroscopy at 30 pulses per second and recorded at 30 frames per second. All stimuli were prepared in 20% w/v barium concentration using E-Z-Paque powdered barium sulfate (Bracco Diagnostics) and bottled water (Nestlé Pure Life) and thickened using a xanthan gum thickener (Resource ThickenUp Clear, Nestlé Health Science). With the thin, slightly thick, and mildly thick liquids, participants were instructed to take comfortable sips and swallow without a cue. With the moderately and extremely thick stimuli, participants were provided with a teaspoon and instructed to take a comfortable spoonful and swallow without a cue. The protocol included three boluses of each consistency, served in blocks of ascending thickness.

### Participants

As reported in the previous article (Waito et al., 2020), participants with a neurologist-confirmed diagnosis of definite–probable ALS using the revised El Escorial criteria (Brooks et al., 2000) were recruited from the University of Florida Norman Fixel Center for Movement Disorders from August 2017 to March 2020. Patients both with and without subjective bulbar symptoms were eligible to participate; however, patients who endorsed bulbar symptoms were preferentially enrolled. Clinical severity of the disease was scored using the Amyotrophic Lateral Sclerosis Functional Rating Scale (ALSFRS; Cedarbaum et al., 1999). Participants were excluded if they had the following:

1. medical history significant for cancer, prior surgery or radiation to the speech or swallowing apparatus;
2. clinical history of dysphagia or a neurological disorder unrelated to ALS;
3. cognitive impairment deemed likely to impair the participant's ability to follow instructions during the study procedures;
4. significant respiratory compromise (e.g., reliance on mechanical ventilation during the day or on a diaphragmatic pacer); or
5. allergies to any of the stimulus ingredients or materials used in data collection.

For the purposes of comparison, data for sex- and age-matched healthy adults were extracted from the healthy control data set collected using the same methods at the Steele Swallowing Lab at the KITE Research Institute–University Health Network in Toronto, Canada (Steele et al., 2023). For each participant with ALS, we identified a matching participant from the healthy data set of the same sex and with the closest age. In the event of multiple eligible matching controls, the participant with the earliest date of data collection was selected. In addition to the exclusion criteria outlined above for PwALS, the healthy participants were eligible, provided that they did not report any history of difficulties with motor speech, gastroesophageal, or neurological function; sinusitis; respiratory or pulmonary disease; or taste disturbance. All participants provided written informed consent before enrollment in the study.

### Videofluoroscopy Rating

Videofluoroscopy rating was performed according to the ASPEKT method, which has been described in detail elsewhere (Steele et al., 2023). The method involves independent duplicate rating of clips for each bolus in two rounds, with consensus resolution of rater discrepancies after each round. The first round begins with identification of the number of swallows per bolus. Next, PAS scores and the integrity of LVC (complete, partial, or incomplete) are documented for each swallow and summarized at the bolus level based on the worst score seen across all swallows for each bolus. Frame numbers are then recorded for a series of key events for the initial swallow of each bolus: BPM, HYB, UES opening, maximum UES distension, UES closure, the first frame of most complete LVC, LVC offset, MPC, and swallow rest. After resolving discrepancies across duplicate ratings for frame identification, timing measures are calculated between events and converted to milliseconds, rounded to the closest frame (i.e., 33-ms increments). These parameters include the following:

- swallow reaction time (i.e., HYB minus BPM);
- the HYB to UES opening interval;
- UES opening duration;
- time-to-most-complete-LVC (LVC minus HYB); and
- LVC duration (LVC offset − LVC).

Key frames are carried forward into the second round of rating, which involves pixel-based tracing of maximum UES opening diameter, pharyngeal area on the frames of MPC and swallow rest, and pharyngeal residue on the frame of swallow rest. All pixel-based measures are normalized to an anatomical scalar defined by the length of the C2–C4 vertebral spine (Molfenter & Steele, 2014). Additionally, for the current analysis, single rater frame-by-frame tracking of hyoid position was performed on all boluses, starting at five frames before onset of the hyoid burst until five frames after arrival at peak hyoid position. The resulting time histories of hyoid position were used to confirm the frame and location of peak hyoid position along the *XY* axis, relative to the anterior–inferior corner of the C4 vertebrae, and to calculate hyoid speed (i.e., change in *XY* position per second, between the frames of HYB and peak *XY* hyoid position). Prior to the analysis for this article, participant mean values were calculated across boluses, by consistency, for the timing and pixel-based parameters. These summarized values were then coded as typical or atypical, relative to reference data and proposed clinical decision limits for healthy adults (Steele et al., 2023). Atypical values were defined as values falling either below the 25th or above the 75th percentile of the healthy reference value distribution, depending on the directionality of the parameter. For example, longer values for swallow reaction time would be considered values of potential clinical concern, and for this parameter, the clinical decision limit was set at the 75th percentile of the healthy reference distribution for each consistency. Conversely, short durations of LVC would be considered of potential clinical concern, and for this parameter, the clinical decision limit was set at the 25th percentile of the healthy reference distribution for each consistency.

## Analysis

Statistical analyses were conducted using SPSS 29.0. Preconsensus interrater reliability was calculated using percent absolute agreement for categorical parameters and intraclass correlations for continuous parameters. Figures illustrating the frequencies of PAS scores (Rosenbek et al., 1996) and the severity of total pharyngeal residue, by consistency, were previously reported in the Waito et al. (2020) study. In this study, we provide additional descriptive statistics regarding overall measures of swallowing safety and efficiency. For Question 1, descriptive statistics (medians, interquartile range) for other quantitative parameters were calculated by cohort and consistency. Mann–Whitney *U* tests were used to identify significant differences between cohorts, within consistency. The criterion for statistical significance was set at *p* < .05. Effect size for the Mann–Whitney *U* tests (*r*) was computed as the ratio between the standardized *Z* statistic and the square root of the number of pairwise comparisons for each test, with values < .3 interpreted as showing a small effect, values from .3 to .5 showing a medium effect size, and values > .5 showing a large effect (Fritz et al., 2012). For Question 2, descriptive statistics for the total number of parameters showing atypical mean values by consistency were tabulated (out of the total possible of 80, i.e., 16 parameters × 5 consistencies), and Mann–Whitney *U* tests with effect size calculations were used to identify differences by cohort, within consistency. Frequency tables for the number of participants showing atypical values were compiled for each parameter by cohort and liquid consistency, and odds ratios for atypical values were calculated, with significant differences defined as odds ratios with a lower 95% confidence interval (CI) value of > 1.

## Results

### Participants

The PwALS cohort comprised 19 adults (nine women and 10 men) with a mean age of 62 years (range: 47–78 years). All 19 PwALS reported race as White; one male participant reported Hispanic ethnicity. The healthy control cohort comprised 19 adults (nine women and 10 men), with a mean age of 64 years (range: 47–82 years). With the exception of two participants of Asian origin, all of the healthy controls reported race as White. All 19 of the healthy controls reported non-Hispanic ethnicity. Table 1 provides a summary of participant demographics for PwALS, including measures of disease severity and duration and information regarding respiratory function.

**Table 1.** Participant demographics for the amyotrophic lateral sclerosis cohort (*n* = 19).

Variable	Bulbar onset	Spinal onset	Mixed onset	Overall
Number of participants, *n* (%)	9 (47%)	9 (47%)	1 (5%)	19
Mean months since diagnosis, *M* (range)	25.7 (9–63)	46.3 (5–96)	10 (n/a)	16.5 (5–96)
Mean months since onset of bulbar symptoms	25.7 (9–63)	12 (0–41)	10 (n/a)	19.6 (0–63)
ALSFRS-R scores				
Total (out of 48), *M* (range)	37 (24–44)	34.3 (23–44)	41 (n/a)	36.5 (23–44)
Bulbar subscale (out of 12), *M* (range)	6.1 (3–9)	10.6 (5–12)	9 (n/a)	8.4 (3–12)
Swallowing subscale (out of 4), *M* (range)	2.6 (1–3)	3.8 (2–4)	3 (n/a)	3.2 (1–4)
Respiratory subscale (out of 12), *M* (range)	10.4 (7–12)	11 (9–12)	11 (n/a)	10.7 (7–12)
Respiratory measures				
FVC (L), *M* (range)	3 (1.4–4.9)	3 (1.1–4.6)	4 (n/a)	3.1 (1.1–4.9)
FVC (%), *M* (range)	74.6% (44–114%)	78% (34–107%)	80% (n/a)	76.6% (34–114%)
FEV1 (L), *M* (range)	2.1 (1.1–3.1)	2.1 (0.5–3.7)	1.9 (n/a)	2.5 (0.5–3.7)
FEV1 (%), *M* (range)	66.4 (45–92)	68.1 (22–99)	64.7 (n/a)	68 (22–99)

*Note.* n/a = not available; ALSFRS-R = Amyotrophic Lateral Sclerosis Functional Rating Scale–Revised; FVC = functional vital capacity; (L) = liters; FEV1 = functional expiratory volume over 1 s.

### Interrater Reliability

As previously reported (Waito et al., 2020), preconsensus categorical ratings of penetration–aspiration and LVC integrity showed perfect agreement in > 75% of cases. For event identification, perfect agreement across raters was seen for 48% of ratings; 92.7% of ratings showed initial discrepancies of ≤ 3 frames. With respect to pixel-based measures, all preconsensus reliability statistics were in the excellent range with intraclass correlation coefficients of > .9. Details regarding preconsensus interrater reliability for ratings of the healthy data set from which the controls were taken can be found in the original publication (Steele et al., 2023).

### Swallowing Safety and Efficiency

Table 2 shows the frequencies for measures of swallowing safety, including worst PAS scores (Rosenbek et al., 1996) and LVC integrity for each cohort, based on the worst score seen for each participant across the repeated boluses of each consistency. In the healthy controls, a worst PAS score of 3 was only seen in a single participant on thin liquids, while scores of 5, 6, 7, and 8 and ratings of partial or incomplete LVC were completely absent. By contrast, frequencies of worst PAS scores of ≥ 3 in PwALS were 47% (TN0), 50% (ST1), 35% (MT2), 31% (MO3), and 25% (EX4). Incomplete LVC was also seen in ≥ 25% of PwALS on all consistencies except extremely thick liquids. Notably, in all cases where aspiration below the true vocal folds without ejection was seen, there was no cough response (i.e., PAS = 8, silent aspiration). Table 3 provides descriptive statistics (median, interquartile range) for measures of swallowing efficiency, including the number of swallows per bolus, total pharyngeal residue, and residue by area (vallecular, pyriform sinus, and elsewhere in the pharynx) by cohort and consistency, based on participant mean values across the repeated boluses of each consistency. Mann–Whitney *U*-test results for cohort comparisons of residue showed significantly higher numbers of swallows per bolus for slightly, mildly, and extremely thick liquid and a single significant finding of greater total residue on extremely thick liquids in PwALS.

**Table 2.** Frequencies for Penetration–Aspiration Scale scores and measures of laryngeal vestibule closure integrity by cohort and consistency.

Cohort	Consistency (IDDSI level)	Worst Penetration–Aspiration Scale score: frequency (%)								Partial or incomplete LVC
		1	2	3	4	5	6	7	8	
PwALS	Thin (TN0)	10 (53)	0 (0)	2 (11)	0 (0)	4 (21)	1 (5)	0 (0)	2 (11)	47%
	Slightly thick (ST1)	8 (44)	1 (6)	2 (11)	0 (0)	4 (22)	0 (0)	0 (0)	3 (17)	50%
	Mildly thick (MT2)	9 (53)	2 (12)	1 (6)	0 (0)	3 (18)	0 (0)	0 (0)	2 (12)	41%
	Moderately thick (MO3)	10 (63)	1 (6)	1 (6)	0 (0)	4 (25)	0 (0)	0 (0)	0 (0)	25%
	Extremely thick (EX4)	12 (75)	2 (13)	1 (6)	0 (0)	1 (6)	0 (0)	0 (0)	0 (0)	6%
Healthy controls	Thin (TN0)	17 (89)	1 (5)	1 (5)	0 (0)	0 (0)	0 (0)	0 (0)	0 (0)	0 (0%)
	Slightly thick (ST1)	18 (95)	1 (5)	0 (0)	0 (0)	0 (0)	0 (0)	0 (0)	0 (0)	0 (0%)
	Mildly thick (MT2)	18 (95)	1 (5)	0 (0)	0 (0)	0 (0)	0 (0)	0 (0)	0 (0)	0 (0%)
	Moderately thick (MO3)	19 (100)	0 (0)	0 (0)	0 (0)	0 (0)	0 (0)	0 (0)	0 (0)	0 (0%)
	Extremely thick (EX4)	19 (100)	0 (0)	0 (0)	0 (0)	0 (0)	0 (0)	0 (0)	0 (0)	0 (0%)

*Note.* IDDSI = International Dysphagia Diet Standardisation Initiative; LVC = laryngeal vestibule closure; PwALS = people with amyotrophic lateral sclerosis.

**Table 3.** Descriptive statistics and cohort comparisons for measures of swallowing efficiency by consistency.

Parameter	Consistency (IDDSI level)	Cohort	*Mdn*	p25	p75	Mann–Whitney *U*	*Z*	*p* (2-tailed)	Effect size	Interpretation
Number of swallows per bolus	Thin (TN0)	PwALS	1	1	1	167	−0.53	.6	0.12	Small
		Healthy controls	1	1	1					
	Slightly thick (ST1)	PwALS	1	1	3	105.5	−2.65	.01*	0.63	Large
		Healthy controls	1	1	1					
	Mildly thick (MT2)	PwALS	1	2	3	93	−2.67	.01*	0.65	Large
		Healthy controls	1	1	1					
	Moderately thick (MO3)	PwALS	1	1	2	123	−1.38	.17	0.35	Medium
		Healthy controls	1	1	1					
	Extremely thick (EX4)	PwALS	1	1	2	104	−2.08	.04*	0.52	Large
		Healthy controls	1	1	1					
Total residue, %(C2–4)^2^	Thin (TN0)	PwALS	1	0	1.8	118	−1.87	.06	0.43	Medium
		Healthy controls	0	0	1					
	Slightly thick (ST1)	PwALS	0.9	0.2	2	131	−1.22	.22	0.29	Small
		Healthy controls	0.7	0	1.6					
	Mildly thick (MT2)	PwALS	0.7	0.1	1.6	145	−0.53	.6	0.13	Small
		Healthy controls	0.8	0	2.3					
	Moderately thick (MO3)	PwALS	1	0.4	1.8	98.5	−1.79	.07	0.45	Medium
		Healthy controls	0.2	0	1.3					
	Extremely thick (EX4)	PwALS	0.8	0.1	1.8	71	−2.73	.01*	0.68	Large
		Healthy controls	0	0	0.6					
Vallecular residue, %(C2–4)^2^	Thin (TN0)	PwALS	0.2	0	1.2	157.5	−0.68	.5	0.16	Small
		Healthy controls	0.4	0	1.2					
	Slightly thick (ST1)	PwALS	0.2	0	1.3	170.5	−0.02	.99	0	None
		Healthy controls	0.7	0	1					
	Mildly thick (MT2)	PwALS	0.1	0	1	140.5	−0.67	.5	0.16	Small
		Healthy controls	0.8	0	1.4					
	Moderately thick (MO3)	PwALS	0.3	0	1.3	139	−0.44	.66	0.11	Small
		Healthy controls	0.2	0	1					
	Extremely thick (EX4)	PwALS	0.4	0	1	128	−0.82	.41	0.2	Small
		Healthy controls	0.2	0	0.8					
Pyriform sinus residue, %(C2–4)^2^	Thin (TN0)	PwALS	0.1	0	0.5	163	−0.53	.6	0.12	Small
		Healthy controls	0.2	0	0.3					
	Slightly thick (ST1)	PwALS	0.1	0	0.4	168	−0.1	.92	0.02	Small
		Healthy controls	0.1	0	0.7					
	Mildly thick (MT2)	PwALS	0.2	0	0.6	157.5	−0.13	.9	0.03	Small
		Healthy controls	0.1	0	0.8					
	Moderately thick (MO3)	PwALS	0.2	0	0.6	116.5	−1.24	.22	0.31	Medium
		Healthy controls	0	0	0.6					
	Extremely thick (EX4)	PwALS	0.1	0	0.4	126	−0.91	.37	0.23	Small
		Healthy controls	0	0	0.4					
Other pharyngeal residue, %(C2–4)^2^	Thin (TN0)	PwALS	0	0	0.1	152	−1.04	.3	0.24	Small
		Healthy controls	0	0	0					
	Slightly thick (ST1)	PwALS	0.2	0	0.4	134	−1.23	.22	0.29	Small
		Healthy controls	0	0	0.5					
	Mildly thick (MT2)	PwALS	0	0	0.1	159	−0.09	.93	0.02	Small
		Healthy controls	0	0	0.3					
	Moderately thick (MO3)	PwALS	0	0	0.4	130	−0.79	.43	0.2	Small
		Healthy controls	0	0	0.2					
	Extremely thick (EX4)	PwALS	0	0	0.5	112	−1.66	.1	0.42	Medium
		Healthy controls	0	0	0					

*Note.* IDDSI = International Dysphagia Diet Standardisation Initiative; PwALS = people with amyotrophic lateral sclerosis.

* *p* < .05.

### Descriptive Statistics for Quantitative VFSS Parameters

**Figure 1.**
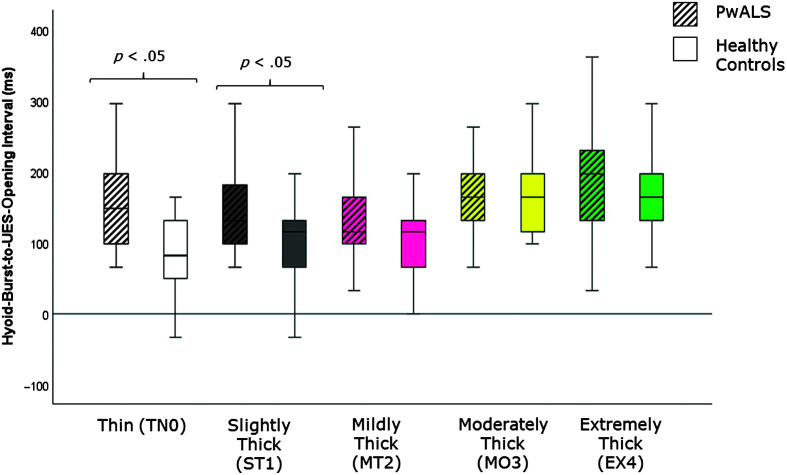
Box plots comparing median and interquartile range values for the hyoid-burst-to-UES-opening interval in PwALS and the age- and sex-matched healthy controls. Significant differences were determined using Mann–Whitney *U* tests. UES = upper esophageal sphincter; PwALS = people with amyotrophic lateral sclerosis.

**Figure 2.**
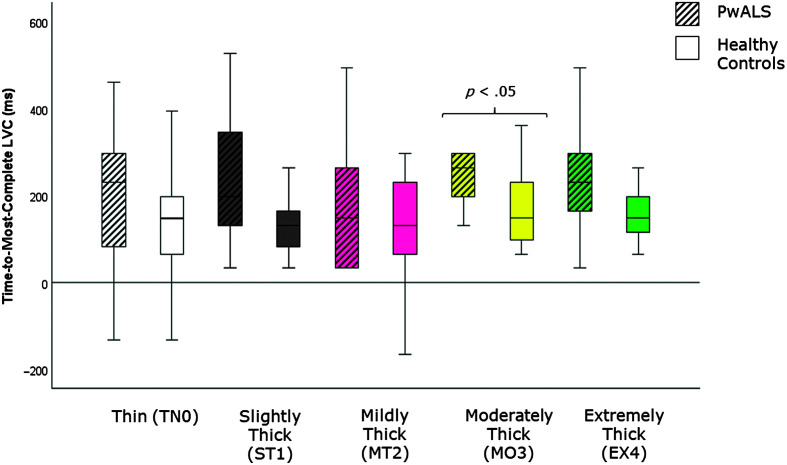
Box plots comparing median and interquartile range values for time-to-most-complete-LVC in PwALS and the age- and sex-matched healthy controls. Significant differences were determined using Mann–Whitney *U* tests. LVC = laryngeal vestibule closure; PwALS = people with amyotrophic lateral sclerosis.

**Figure 3.**
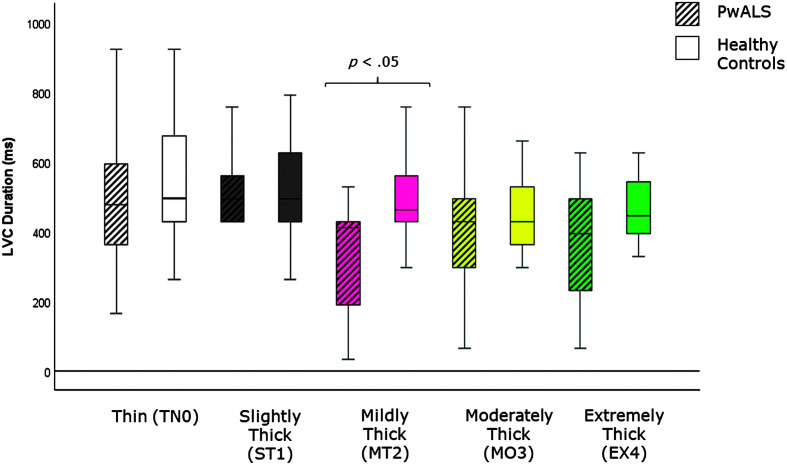
Box plots comparing median and interquartile range values for LVC duration in PwALS and the age- and sex-matched healthy controls. Significant differences were determined using Mann–Whitney *U* tests. LVC = laryngeal vestibule closure; PwALS = people with amyotrophic lateral sclerosis.

**Figure 4.**
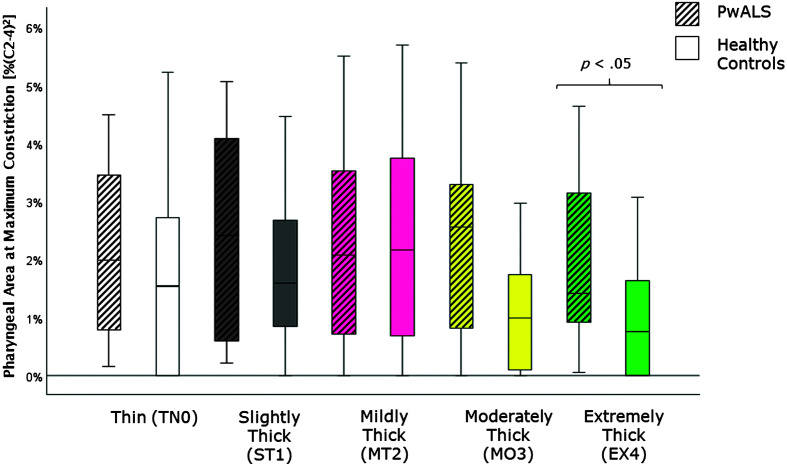
Box plots comparing median and interquartile range values for pharyngeal area at maximum constriction in PwALS and the age- and sex-matched healthy controls. Significant differences were determined using Mann–Whitney *U* tests. PwALS = people with amyotrophic lateral sclerosis.

**Figure 5.**
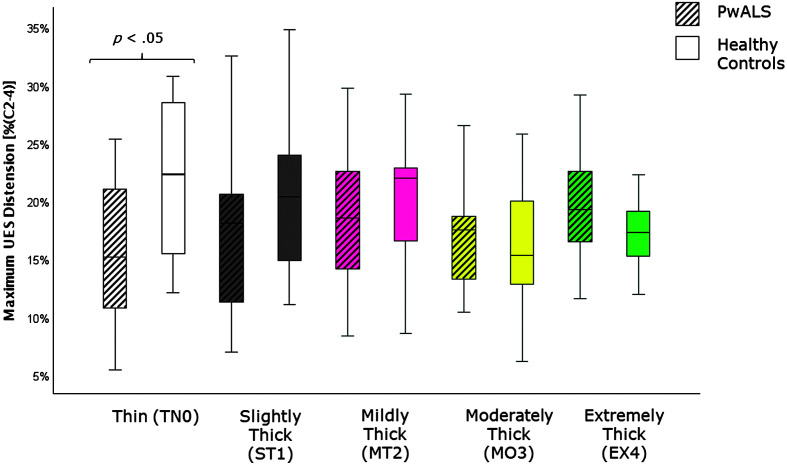
Box plots comparing median and interquartile range values for maximum UES distension in PwALS and the age- and sex-matched healthy controls. Significant differences were determined using Mann–Whitney *U* tests. UES = upper esophageal sphincter; PwALS = people with amyotrophic lateral sclerosis.

**Figure 6.**
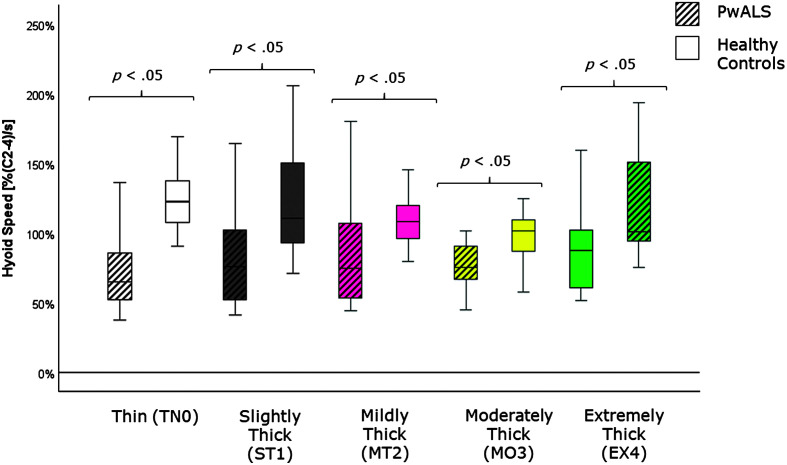
Box plots comparing median and interquartile range values for hyoid speed in PwALS and the age- and sex-matched healthy controls. Significant differences were determined using Mann–Whitney *U* tests. PwALS = people with amyotrophic lateral sclerosis.

Table 4 provides descriptive statistics for each quantitative VFSS parameter, by consistency, based on participant mean values across the repeated boluses of each consistency. The results of the Mann–Whitney *U* tests can be found on the right-hand side of this table together with the effect size statistics and interpretation. Several parameters did not show significant differences compared to the age-matched controls. These included swallow reaction time (all consistencies); the hyoid-burst-to-UES-opening interval (MT2, MO3, EX4); UES opening duration (TN0, ST1, MT2); time-to-most-complete-LVC (all consistencies except MO3); LVC duration (all consistencies except MT2); pharyngeal constriction (all consistencies except EX4); UES distension (all consistencies except TN0); peak *XY* hyoid position (all consistencies); the number of swallows per bolus (TN0, MO3); vallecular, pyriform sinus, and other pharyngeal residue (all consistencies); and total pharyngeal residue (TN0, ST1, MT2, MO3). With respect to significant differences in the direction of clinical concern for timing measures, these analyses showed consistency-specific findings of significantly longer hyoid-burst-to-UES-opening interval duration (TN0, ST1), significantly longer time-to-most-complete-LVC (MO3), and significantly shorter LVC duration (MT2) in PwALS. These findings are illustrated using box plots in Figures 1–3. Pixel-based measures showed significantly worse pharyngeal constriction (i.e., larger area) on extremely thick liquids and confirmed the previously reported finding of significantly narrower UES distension on thin liquids (see Figures 4 and 5). Finally, as illustrated in Figure 6, significantly reduced hyoid speed was seen in PwALS for all consistencies.

**Table 4.** Descriptive statistics and cohort comparisons for quantitative videofluoroscopic swallowing studies measures of swallow timing and kinematics by consistency.

Parameter	Consistency (IDDSI level)	Cohort	*Mdn*	p25	p75	Mann–Whitney *U*	*Z*	*p* (2-tailed)	Effect size	Interpretation
Swallow reaction time (ms)	Thin (TN0)	PwALS	149	66	363	152.5	−0.29	.77	0.07	Small
		Healthy controls	231	99	330					
	Slightly thick (ST1)	PwALS	396	165	825	117	−1.64	.1	0.38	Medium
		Healthy controls	264	99	429					
	Mildly thick (MT2)	PwALS	413	182	528	122	−1	.32	0.24	Small
		Healthy controls	330	99	495					
	Moderately thick (MO3)	PwALS	297	116	627	125.5	−0.27	.78	0.07	Small
		Healthy controls	231	165	594					
	Extremely thick (EX4)	PwALS	545	191	1295	121.5	−1.01	.31	0.25	Small
		Healthy controls	462	66	759					
Hyoid-burst-to-UES-opening interval (ms)	Thin (TN0)	PwALS	132	99	198	68.5	−3.32	.00*	0.76	Large
		Healthy controls	99	33	132					
	Slightly thick (ST1)	PwALS	132	99	182	96.5	−2.3	.02*	0.54	Large
		Healthy controls	132	66	132					
	Mildly thick (MT2)	PwALS	132	99	190	139	−0.72	.47	0.18	Small
		Healthy controls	132	66	132					
	Moderately thick (MO3)	PwALS	165	132	215	148.5	−0.12	.91	0.03	Small
		Healthy controls	165	132	198					
	Extremely thick (EX4)	PwALS	198	132	231	135	−0.57	.57	0.14	Small
		Healthy controls	165	132	198					
UES opening duration (ms)	Thin (TN0)	PwALS	545	429	594	162.5	−0.53	.6	0.12	Small
		Healthy controls	495	462	561					
	Slightly thick (ST1)	PwALS	528	495	627	124	−1.44	.15	0.34	Medium
		Healthy controls	495	429	561					
	Mildly thick (MT2)	PwALS	545	470	594	127.5	−1.09	.28	0.26	Small
		Healthy controls	495	429	528					
	Moderately thick (MO3)	PwALS	495	454	528	39.5	−3.77	.00*	0.94	Large
		Healthy controls	396	363	429					
	Extremely thick (EX4)	PwALS	495	437	528	68	−2.82	.00*	0.7	Large
		Healthy controls	396	396	462					
Time-to-most-complete-LVC (ms)	Thin (TN0)	PwALS	182	33	231	122.5	−1.48	.14	0.35	Medium
		Healthy controls	165	66	198					
	Slightly thick (ST1)	PwALS	165	66	347	125.5	−1.39	.16	0.33	Medium
		Healthy controls	132	99	165					
	Mildly thick (MT2)	PwALS	149	41	264	156	−0.18	.86	0.04	Small
		Healthy controls	132	66	231					
	Moderately thick (MO3)	PwALS	264	182	338	74	−2.6	.01*	0.65	Large
		Healthy controls	165	99	231					
	Extremely thick (EX4)	PwALS	231	116	297	99	−1.77	.08	0.44	Medium
		Healthy controls	165	132	198					
LVC duration (ms)	Thin (TN0)	PwALS	495	347	644	147	−0.46	.64	0.11	Small
		Healthy controls	495	429	693					
	Slightly thick (ST1)	PwALS	495	314	594	152	−0.3	.76	0.07	Small
		Healthy controls	495	429	627					
	Mildly thick (MT2)	PwALS	429	314	454	87	−2.38	.02*	0.58	Large
		Healthy controls	462	429	594					
	Moderately thick (MO3)	PwALS	429	264	512	116.5	−1.18	.24	0.3	Medium
		Healthy controls	429	363	528					
	Extremely thick (EX4)	PwALS	413	231	520	113.5	−1.28	.2	0.32	Medium
		Healthy controls	462	396	561					
Pharyngeal area at maximum constriction, %(C2–4)^2^	Thin (TN0)	PwALS	1.7	0.4	3.1	148	−0.95	.34	0.22	Small
		Healthy controls	1.8	0	2.8					
	Slightly thick (ST1)	PwALS	2.4	0.6	4.1	126	−1.37	.17	0.32	Medium
		Healthy controls	1.6	0.5	3.2					
	Mildly thick (MT2)	PwALS	2.1	0.7	3.5	154	−0.24	.81	0.06	Small
		Healthy controls	2.1	0.5	3.8					
	Moderately thick (MO3)	PwALS	2.4	0.4	3.2	92.5	−1.98	.05	0.49	Medium
		Healthy controls	1	0	1.9					
	Extremely thick (EX4)	PwALS	2	0.6	3.2	88	−2.12	.03*	0.53	Large
		Healthy controls	0.8	0	1.8					
Maximum UES distension, %(C2–4)	Thin (TN0)	PwALS	18.2	12.5	22.6	101	−2.32	.02*	0.53	Large
		Healthy controls	21.9	15	28.3					
	Slightly thick (ST1)	PwALS	19.5	11.3	21.4	134.5	−1.11	.27	0.26	Small
		Healthy controls	19.8	13.5	25.7					
	Mildly thick (MT2)	PwALS	20	14.5	24.6	138	−0.74	.46	0.18	Small
		Healthy controls	21.9	16	22.7					
	Moderately thick (MO3)	PwALS	17.8	14.4	20.9	134	−0.6	.55	0.15	Small
		Healthy controls	15.4	12.7	20.4					
	Extremely thick (EX4)	PwALS	20	16	24.7	101.5	−1.67	.09	0.42	Small
		Healthy controls	16.7	14.8	19.7					
Hyoid peak *XY* position, %(C2–4)	Thin (TN0)	PwALS	150	163.9	179.8	112	−1.27	.21	0.29	Small
		Healthy controls	157.7	173	187					
	Slightly thick (ST1)	PwALS	151.6	162.8	180.2	120	−1.06	.29	0.25	Small
		Healthy controls	156.3	172	184.5					
	Mildly thick (MT2)	PwALS	153.6	171	176.7	123	−0.99	.32	0.24	Small
		Healthy controls	159.2	172.7	183					
	Moderately thick (MO3)	PwALS	153.6	166.9	180.1	116	−0.72	.47	0.18	Small
		Healthy controls	155.1	172.7	181.5					
	Extremely thick (EX4)	PwALS	157.1	168.6	183.1	113	−0.83	.41	0.21	Small
		Healthy controls	158.4	172.8	183.9					
Hyoid speed, %(C2–4)/s	Thin (TN0)	PwALS	50.9	65.4	86.7	40	−3.67	.00*	0.84	Large
		Healthy controls	105.3	122.7	146.3					
	Slightly thick (ST1)	PwALS	52.4	75.9	108.7	69	−2.75	.01*	0.65	Large
		Healthy controls	92.8	111.2	152.4					
	Mildly thick (MT2)	PwALS	52.2	74.8	108.3	69	−2.77	.01*	0.67	Large
		Healthy controls	93.8	108.3	122					
	Moderately thick (MO3)	PwALS	65.1	75.8	96.1	76	−2.16	.03*	0.54	Large
		Healthy controls	86.4	101.8	110.3					
	Extremely thick (EX4)	PwALS	59.7	87.7	103	67	−2.49	.01*	0.62	Large
		Healthy controls	93.7	101.2	154					

*Note.* IDDSI = International Dysphagia Diet Standardisation Initiative; PwALS = people with amyotrophic lateral sclerosis.

* *p* < .05.

### Frequency of Atypical Values

**Figure 7.**
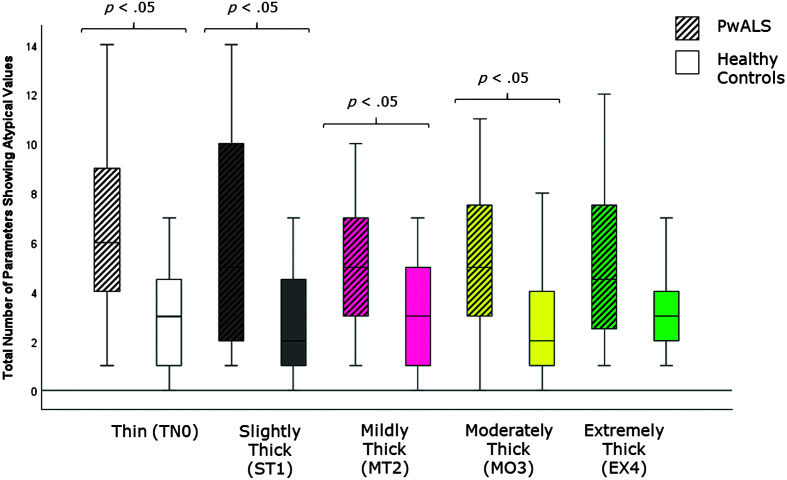
Box plots comparing median and interquartile range values for the number of parameters showing atypical values in PwALS and the age- and sex-matched healthy controls. Significant differences were determined using Mann–Whitney *U* tests. PwALS = people with amyotrophic lateral sclerosis.

Table 5 shows descriptive statistics (median, interquartile range) for the total number of atypical values seen across the 16 parameters and five consistencies by cohort and consistency. PwALS showed a higher number of atypical values (overall estimated marginal mean = 5.87, 95% CI [4.59, 7.15]) than the healthy controls (overall estimated marginal mean = 3.04, 95% CI [1.77, 4.31]), and the Mann–Whitney *U* tests confirmed this difference to be significant for all consistencies except extremely thick liquids, as illustrated in Figure 7. Table 6 shows participant frequencies of atypical values for each parameter for PwALS compared to age-matched healthy controls. Odds ratios for higher frequencies of atypical values in the ALS cohort are shown, with 95% CI boundaries. PwALS showed significantly higher frequencies of atypical values as follows:

**Table 5.** Descriptive statistics and cohort comparisons for the total number of atypical parameters by consistency.

Consistency (IDDSI level)	Cohort	*Mdn*	p25	p75	Mann–Whitney *U*	*Z*	*p* (2-tailed)
Thin (TN0)	PwALS	6.0	4.0	9.0	76.5	−3.05	< .01*
	Healthy controls	3.0	1.0	5.0			
Slightly thick (ST1)	PwALS	5.0	2.0	10.0	88.5	−2.53	.01*
	Healthy controls	2.0	1.0	5.0			
Mildly thick (MT2)	PwALS	5.0	3.0	7.5	99.5	−1.98	.05
	Healthy controls	3.0	1.0	5.0			
Moderately thick (MO3)	PwALS	5.0	3.0	7.8	87.5	−2.15	.03*
	Healthy controls	2.0	1.0	4.0			
Extremely thick (EX4)	PwALS	4.5	2.3	7.8	105.5	−1.56	.12
	Healthy controls	3.0	2.0	4.0			

*Note.* IDDSI = International Dysphagia Diet Standardisation Initiative; PwALS = people with amyotrophic lateral sclerosis.

* *p* < .05.

**Table 6.** Frequencies and odds ratios for atypical values by cohort and consistency.

Parameter	Consistency (IDDSI level)	Definition of atypical values^a^	PwALS %typical	Healthy controls %atypical	Odds ratio	95% CI boundaries
Maximum Penetration–Aspiration Scale score	Thin (TN0)	≥ 2	47	5	17.2	[1.79, 147.07]
	Slightly thick (ST1)	≥ 2	50	0	21.1^b^	[2.33, 191.17]
	Mildly thick (MT2)	≥ 2	35	0	12.09^b^	[1.31, 111.66]
	Moderately thick (MO3)	≥ 2	31	0	10.4^b^	[1.1, 97.7]
	Extremely thick (EX4)	≥ 2	13	0	*n.s.*	
LVC integrity	Thin (TN0)	Partial or incomplete	47	0	17.3^b^	[1.94, 153.67]
	Slightly thick (ST1)	Partial or incomplete	50	0	21.1^b^	[2.33, 191.17]
	Mildly thick (MT2)	Partial or incomplete	41	0	15.2^b^	[1.66, 139.31]
	Moderately thick (MO3)	Partial or incomplete	25	0	7.9^b^	[0.82, 76.28]
	Extremely thick (EX4)	Partial or incomplete	6	0	*n.s.*	
Swallows per bolus	Thin (TN0)	≥ 2	26	21	*n.s.*	
	Slightly thick (ST1)	≥ 2	44	5	14.4	[1.57, 132.31]
	Mildly thick (MT2)	≥ 2	53	11	9.6	[1.67, 54.89]
	Moderately thick (MO3)	≥ 2	31	11	*n.s.*	
	Extremely thick (EX4)	≥ 2	44	11	6.6	[1.13, 38.7]
Total residue, %(C2–4)^2^	Thin (TN0)	> 1.7	32	21	*n.s.*	
	Slightly thick (ST1)	> 1.9	33	21	*n.s.*	
	Mildly thick (MT2)	> 2.2	18	26	*n.s.*	
	Moderately thick (MO3)	> 2.1	38	16	*n.s.*	
	Extremely thick (EX4)	> 1.5	25	5	*n.s.*	
Vallecular residue, %(C2–4)^2^	Thin (TN0)	> 0.7	42	37	*n.s.*	
	Slightly thick (ST1)	> 1.0	33	21	*n.s.*	
	Mildly thick (MT2)	> 1.1	18	32	*n.s.*	
	Moderately thick (MO3)	> 0.6	44	26	*n.s.*	
	Extremely thick (EX4)	> 0.5	44	37	*n.s.*	
Pyriform sinus residue, %(C2–4)^2^	Thin (TN0)	> 0.5	26	16	*n.s.*	
	slightly thick (ST1)	> 0.6	17	26	*n.s.*	
	Mildly thick (MT2)	> 0.4	41	37	*n.s.*	
	Moderately thick (MO3)	> 0.5	25	26	*n.s.*	
	Extremely thick (EX4)	> 0.5	6	16	*n.s.*	
Other pharyngeal residue, %(C2–4)^2^	Thin (TN0)	> 0.3	21	16	*n.s.*	
	Slightly thick (ST1)	> 0.3	28	26	*n.s.*	
	Mildly thick (MT2)	> 0.6	12	11	*n.s.*	
	Moderately thick (MO3)	> 0.6	13	5	*n.s.*	
	Extremely thick (EX4)	> 0.6	19	11	*n.s.*	
Swallow reaction time (ms)	Thin (TN0)	> 400	18	16	*n.s.*	
	Slightly thick (ST1)	> 400	44	32	*n.s.*	
	Mildly thick (MT2)	> 534	19	21	*n.s.*	
	Moderately thick (MO3)	> 667	21	21	*n.s.*	
	Extremely thick (EX4)	> 801	38	21	*n.s.*	
Hyoid-burst-to-UES-opening interval (ms)	Thin (TN0)	> 133	53	5	20	[2.2, 181.56]
	Slightly thick (ST1)	> 167	28	5	*n.s.*	
	Mildly thick (MT2)	> 167	24	11	*n.s.*	
	Moderately thick (MO3)	> 200	25	16	*n.s.*	
	Extremely thick (EX4)	> 200	31	16	*n.s.*	
UES opening duration (ms)	Thin (TN0)	< 434	32	21	*n.s.*	
	Slightly thick (ST1)	< 400	11	16	*n.s.*	
	Mildly thick (MT2)	< 400	24	16	*n.s.*	
	Moderately thick (MO3)	< 367	0	26	*n.s.*	
	Extremely thick (EX4)	< 367	6	21	*n.s.*	
Time-to-most-complete-LVC (ms)	Thin (TN0)	> 167	58	32	*n.s.*	
	Slightly thick (ST1)	> 234	39	16	*n.s.*	
	Mildly thick (MT2)	> 200	35	32	*n.s.*	
	Moderately thick (MO3)	> 200	69	32	4.8	[1.14, 19.98]
	Extremely thick (EX4)	> 167	69	37	*n.s.*	
LVC duration (ms)	Thin (TN0)	< 400	29	16	*n.s.*	
	Slightly thick (ST1)	< 400	24	21	*n.s.*	
	Mildly thick (MT2)	< 400	47	21	*n.s.*	
	Moderately thick (MO3)	< 400	44	42	*n.s.*	
	Extremely thick (EX4)	< 400	50	32	*n.s.*	
Maximum UES distension, %(C2–4)	Thin (TN0)	< 17	53	33	*n.s.*	
	Slightly thick (ST1)	< 15	33	26	*n.s.*	
	Mildly thick (MT2)	< 15	29	16	*n.s.*	
	Moderately thick (MO3)	< 12	13	11	*n.s.*	
	Extremely thick (EX4)	< 14	13	16	*n.s.*	
Pharyngeal area at Maximum pharyngeal constriction, %(C2–4)^2^	thin (TN0)	> 2.7	42	26	*n.s.*	
	Slightly thick (ST1)	> 2.5	50	26	*n.s.*	
	Mildly thick (MT2)	> 3.3	29	32	*n.s.*	
	Moderately thick (MO3)	> 2.1	69	16	11.7	[2.31, 59.54]
	Extremely thick (EX4)	> 1.4	56	32	*n.s.*	
Hyoid peak *XY* position, %(C2–4)	Thin (TN0)	< 163	50	27	*n.s.*	
	Slightly thick (ST1)	< 159	42	25	*n.s.*	
	Mildly thick (MT2)	< 161	39	29	*n.s.*	
	Moderately thick (MO3)	< 158	29	31	*n.s.*	
	Extremely thick (EX4)	< 160	29	25	*n.s.*	
Hyoid speed, %(C2–4)/s	Thin (TN0)	< 103	85	13	36.8	[5.35, 253.62]
	Slightly thick (ST1)	< 94	68	25	6.5	[1.47, 28.8]
	Mildly thick (MT2)	< 96	72	24	8.5	[1.84, 38.75]
	Moderately thick (MO3)	< 89	65	31	*n.s.*	
	Extremely thick (EX4)	< 89	53	13	7.88	[1.35, 45.83]

*Note.* IDDSI = International Dysphagia Diet Standardisation Initiative; PwALS = people with amyotrophic lateral sclerosis; CI = confidence interval; *n.s.* = nonsignificant; LVC = laryngeal vestibule closure.

a Steele et al. (2023).

b Haldane–Anscombe correction for zero cell counts applied.

- PAS scores of 2 or greater on all consistencies except extremely thick liquids;
- partial or incomplete LVC on all consistencies except extremely thick liquids;
- multiple swallows per bolus on slightly and mildly thick liquids;
- a prolonged hyoid-burst-to-UES opening interval on thin liquids;
- prolonged time-to-most-complete-LVC on moderately thick liquids;
- poor pharyngeal constriction represented by a large pharyngeal area at maximum constriction on moderately thick liquids; and
- reduced hyoid speed on all consistencies except moderately thick liquids.

## Discussion

This study adds to our knowledge regarding the nature of swallowing impairment in PwALS. Overall, the results are in agreement with previous studies employing videofluoroscopy, which have reported high frequencies of penetration–aspiration, particularly on thin liquids (Higo et al., 2004; Park et al., 2022; Robison et al., 2022, 2023; Waito et al., 2020). The results are also concordant with previous studies showing that reduced pharyngeal constriction and inefficiency in the form of pharyngeal residue and multiple swallows per bolus are common in PwALS (Donohue et al., 2023; Higo et al., 2004; Park et al., 2022; Robison et al., 2022, 2023; Waito et al., 2020). It should be noted that differences in study methods, including the properties (consistency, volume, radio-opacity) and number of stimuli used, and in parameter definitions and analysis methods mean that results syntheses across studies should be made with caution.

The current study builds specifically on our previous analysis of quantitative measures of swallowing with thin liquids (Waito et al., 2020) by expanding the range of liquid consistencies studied from thin to extremely thick liquids. Here, we can appreciate that some of the cohort differences that are seen on thin liquids do not occur with thicker consistencies. Specifically, a prolonged hyoid-burst-to-UES-opening interval was seen in PwALS on thin and slightly thick liquids, but was not apparent with mildly, moderately, or extremely thick liquids. Similarly, reduced UES opening duration in PwALS was only seen on thin liquids. Conversely, thicker liquids revealed some cohort differences that were not visible on thin liquids. These included long time-to-most-complete-LVC (MO3), short durations of UES opening (MO3, EX4) and LVC (MT2), larger pharyngeal area at maximum constriction (EX4), and greater total residue (EX4) in PwALS.

The previous analysis by Waito et al. (2020) identified longer durations of LVC and UES opening as features of thin liquid swallowing in PwALS suggestive of potential compensation. Notably, findings of prolonged UES opening and LVC durations on thin liquids have been identified as an age-related change in swallowing in the larger reference sample (Mancopes et al., 2021). Therefore, it is likely that the previously reported cohort differences for these parameters are attributable to the fact that comparisons were made to a younger reference sample. The current analysis confirmed a finding of significantly longer UES opening durations in PwALS, but rather than this phenomenon being seen with thin liquids, it was seen on the moderately and extremely thick consistencies. Prolonged LVC durations were not seen in PwALS in the current analysis.

A novel contribution of this study is the classification of the data as typical or atypical relative to reference data for healthy adults aged 21–82 years, collected using the same data collection protocol and analyzed using the same methods (Steele et al., 2023). The available reference data span a larger age range than those that were available for the Waito et al. (2020) study. Additionally, the current analysis uses different clinical decision limits, set at either the 75th or 25th percentile of the healthy reference distribution (depending on the directionality of the parameter), as proposed by Steele et al. (2023). These thresholds are less extreme than the ±2 *SD* or 95th percentile thresholds used in other studies; this shift impacts the degree to which findings of potential clinical concern appear to be common in PwALS (Robison et al., 2022, 2023; Waito et al., 2020).

In addition to examining parameter values relative to the healthy reference distribution, we have controlled for the potential influence of advancing age to the presentation of swallowing in PwALS through comparative analysis of the frequency of atypical values in PwALS and age- and sex-matched healthy controls. Here, the analysis shows overall that PwALS showed a significantly higher number of atypical findings than the age-matched healthy controls. In our previous analysis of the thin liquid swallows from the same data set (Waito et al., 2020), we identified significantly increased frequencies of partial or incomplete LVC, prolonged time-to-LVC, narrow UES distension, and poor pharyngeal constriction in PwALS as mechanistic factors associated with impaired swallowing safety and efficiency. Despite shifts in the clinical decision limits used for classifying the data as typical or atypical, the current analysis confirmed partial or incomplete LVC to be a prominent finding in PwALS, associated with impaired swallowing safety on thin, slightly thick, and mildly thick consistencies. The results also confirmed a pattern of multiple swallows per bolus as a prominent characteristic in PwALS across multiple consistencies.

The cohort comparisons of parameter values for measures of swallow timing and kinematics in this study revealed several consistency-specific differences, which were largely (but not completely) congruent with increased odds of atypical values for the same parameter and consistency combination. The fact that these results were seen on only one or two consistencies per parameter, together with the small sample size in this study, suggests that these findings are not sufficiently pervasive to be considered characteristics of swallowing in ALS. One exception was the prominent difference identified between PwALS and age-matched healthy controls in hyoid speed, which was reduced below the atypical/typical boundary across all consistencies in PwALS. The speed of hyoid movement has been linked to the goal of achieving LVC (Nagy et al., 2014); thus, the correspondence between findings of partial/incomplete LVC and reduced hyoid speed in PwALS may identify a particular mechanism contributing to impaired swallowing safety in this group as well as targets for compensatory or rehabilitative intervention.

Comparisons of the frequency of atypical parameter values across cohorts also means that relatively high frequencies of atypical findings in the healthy control cohort can mitigate the degree to which certain findings appear to be characteristics of ALS. In this respect, the finding that the frequency of atypical pharyngeal residue did not differ between PwALS and healthy controls is the most suprising, given previous reports of atypical residue being a prominent concern in PwALS (Donohue et al., 2023; Robison et al., 2022, 2023). When comparing these studies, several methodological differences must be acknowledged, beginning with the fact that the University of Florida studies did not include comparisons of residue severity to age-matched controls. In our analysis, the Mann–Whitney *U* tests confirmed worse residue in PwALS on the thin and moderately thick liquid consistencies. However, ≥ 16% of the age-matched healthy controls also showed residue above the clinical decision limit on all consistencies except extremely thick liquids. Several studies suggest that poor pharyngeal constriction is a primary mechanism behind residue accumulation (Stokely et al., 2015; Waito et al., 2018) and that pharyngeal constriction deteriorates with advancing age (Kendall & Leonard, 2001c; Leonard et al., 2004; Mancopes et al., 2021; Molfenter et al., 2019, 2020). Therefore, it seems likely that the presence of residue in PwALS is at least partly attributable to age-related changes.

When comparing data across these studies, it is important to acknowledge other methodological differences that may have influenced the results with respect to the prevalence of pharyngeal residue. These include the use of different barium stimuli, namely, Bracco Varibar 40% w/v concentration barium sulfate in thin, thin honey, and pudding consistencies in the Robison et al. and Donohue et al. studies and 20% w/v concentration Bracco E-Z-Paque barium thickened with Nestlé's Resource ThickenUp Clear xanthan gum thickener in this study. Examination of the ALSFRS-R scores also suggests that the patients in the Robison et al. and Donohue et al. studies were at a more advanced disease stage than those in this study, with this observation supported by data showing higher frequencies of unsafe swallowing and worse scores for the number of swallows per bolus. Differences in the conventions used for deriving total pharyngeal residue scores and in the thresholds used for defining values of concern across studies must also be noted. In our study, total pharyngeal residue was calculated as the sum of residue area in the valleculae, pyriform sinuses, and elsewhere in the pharynx at the end of the initial swallow for each bolus. Participant mean values for total residue were then calculated across three bolus repetitions per consistency, and these were used for determining whether residue severity crossed the clinical decision limit. By contrast, in the Robison et al. studies, protocol-level participant worst scores for vallecular residue, pyriform sinus residue, and other pharyngeal residue were identified across three bolus conditions (5 cc boluses of thin liquid barium, cup sips of thin liquid barium, and 5 cc teaspoon boluses of moderately thick barium). These protocol-level worst scores, which may have been drawn from different boluses for each residue location, were then summed for an overall estimate of worst total pharyngeal residue, and inefficiency was defined as a worst value of ≥ 3% (C2–4)^2^. Given these differences in summation, direct comparison of measures across studies is questionably appropriate.

### Limitations

This study was not without its limitations. First, the data were collected from a cross-sectional sample at a single time point and, therefore, do not shed light on the progression of swallowing impairment in ALS. Second, we recruited a heterogeneous cohort of PwALS, both with respect to onset pattern and the presence of reported bulbar symptoms at the time of enrollment but excluding individuals with more advanced respiratory symptoms. The resulting sample turned out to have relatively mild disease, as indicated by their mean ALSFRS-R total score. Therefore, it is unclear how these results would generalize to other ALS patients with differing disease severities. Future longitudinal research comparing patients at the mild, moderate, and severe stages of disease is necessary to better understand how these swallowing impairment profiles might change based on disease severity. Third, to summarize results across multiple repetitions for each task and consistency, the “worst” PAS scores and mean values for other parameters were captured per bolus consistency for each participant. As highlighted in the discussion section, these conventions may differ from those used in other studies and do not account for the variation or frequency of specific scores seen across boluses during a VFSS.

A major limitation of this study lies in the small sample size of 19 PwALS, which certainly does not allow for the exploration of additional factors of interest such as disease onset type and severity. The data were collected as part of a larger grant, for which power calculations originally suggested that a sample of 40 participants would be the minimum required to identify minimally interesting differences in any single parameter. We were only successful in recruiting half of that target number within the study time frame. The power calculation was conducted using previously available data for measures of maximum isometric tongue pressure rather than quantitative videofluoroscopy data. We acknowledge that the decision to perform analyses within consistency, without adjustment of the alpha level for multiple comparisons, may increase the risk of Type I error. The decision to use participant mean values per consistency across repetitions neutralizes the impact of extreme values and mitigates this concern to some degree. We also did not adjust the alpha level for possible auto-correlations across parameters. Given these choices, the apparent significance of results for any one parameter should be considered cautiously in the broader context of the overall results, and findings that appear to occur systematically across multiple consistencies should be considered more robust than those occurring on single consistencies.

## Conclusions

In summary, this extended analysis of VFSS data from a cohort of 19 adults with ALS, across the full range of liquid consistencies, and compared to age- and sex-matched healthy controls clarifies the nature of changes in swallowing that are seen in ALS. Changes seen across consistencies include impairments in LVC with associated penetration–aspiration, inefficiency in the form of multiple swallows per bolus, and reduced hyoid speed. These impairments were noted in the context of and in addition to changes that appear to be attributable to aging, including reduced pharyngeal constriction and associated pharyngeal residue. Given the relatively mild disease stage of PwALS in this study, future longitudinal studies would be valuable for elucidating how changes in swallowing emerge across the course of disease progression.

## Author Contributions

**Pooja Gandhi:** Formal analysis (Supporting), Writing – original draft (Equal). **Ashley A. Waito:** Investigation (Supporting), Data curation (Lead), Writing – review & editing (Supporting). **Melanie Peladeau-Pigeon:** Data curation (Supporting), Investigation (Supporting), Project administration (Supporting), Software (Lead), Validation (Lead), Writing – review & editing (Supporting). **Emily K. Plowman:** Resources (Supporting), Supervision (Supporting), Writing – review & editing (Supporting). **Catriona M. Steele:** Conceptualization (Lead), Formal analysis (Lead), Funding acquisition (Lead), Investigation (Lead), Methodology (Lead), Project administration (Lead), Resources (Lead), Supervision (Lead), Visualization (Lead), Writing – original draft (Equal).

## Data Availability Statement

The data sets generated during and/or analyzed during the current study are not publicly available due to ethical/legal restrictions. Inquiries regarding access to the data should be directed to the corresponding author.
